# Aortic Regurgitation After Right Coronary Cusp Injury During Percutaneous Coronary Intervention

**DOI:** 10.7759/cureus.52560

**Published:** 2024-01-19

**Authors:** Taylor Bowman, Donal O'Donoghue, Jose L Diz Ferre, Leonardo A Marquez Roa, Richard Hofstra, Sabry Ayad

**Affiliations:** 1 Anesthesiology, Cleveland Clinic South Pointe Hospital, Cleveland, USA; 2 Anesthesiology, Cleveland Clinic Fairview Hospital, Cleveland, USA; 3 Outcomes Research, Cleveland Clinic, Cleveland, USA

**Keywords:** surgical aortic valve replacement (savr), transesophageal echocardiogram (tee), left heart catheterization, right coronary cusp injury, iatrogenic aortic regurgitation

## Abstract

Injury of a coronary cusp of the aortic valve is a rare complication that can occur during coronary angiography. It usually occurs from multiple attempts with different catheters to access the ostia of the right coronary artery, but it has also occurred accessing the ostia of the left coronary artery. We present the case of a patient who underwent coronary angiography with suspected coronary cusp injury that remained asymptomatic but was found to have severe aortic regurgitation during coronary artery bypass graft surgery (CABG) one week later, requiring an aortic valve replacement.

## Introduction

Coronary angiography is a diagnostic and possible therapeutic procedure to identify blockages in the coronary arteries, commonly conducted during or after myocardial infarction. The technique consists of inserting a catheter through either the radial or femoral artery, navigating it through the aorta, and positioning it near the coronary cusps close to the aortic valve [1]. Then, the catheter is directed into either the right or left coronary artery while injecting dye to assess the presence of blockages to determine the necessary treatment. In cases of severe blockage or multivessel disease, patients are usually referred for coronary artery bypass graft surgery (CABG). During CABG, the surgeons harvest vessels, often from the left internal mammary artery (LIMA), saphenous vein, or radial artery [2], and graft them onto the affected coronary arteries. This procedure bypasses the obstructed portions of the coronary arteries and reperfuses the myocardial tissue beyond the blockage.

## Case presentation

**Figure 1:**
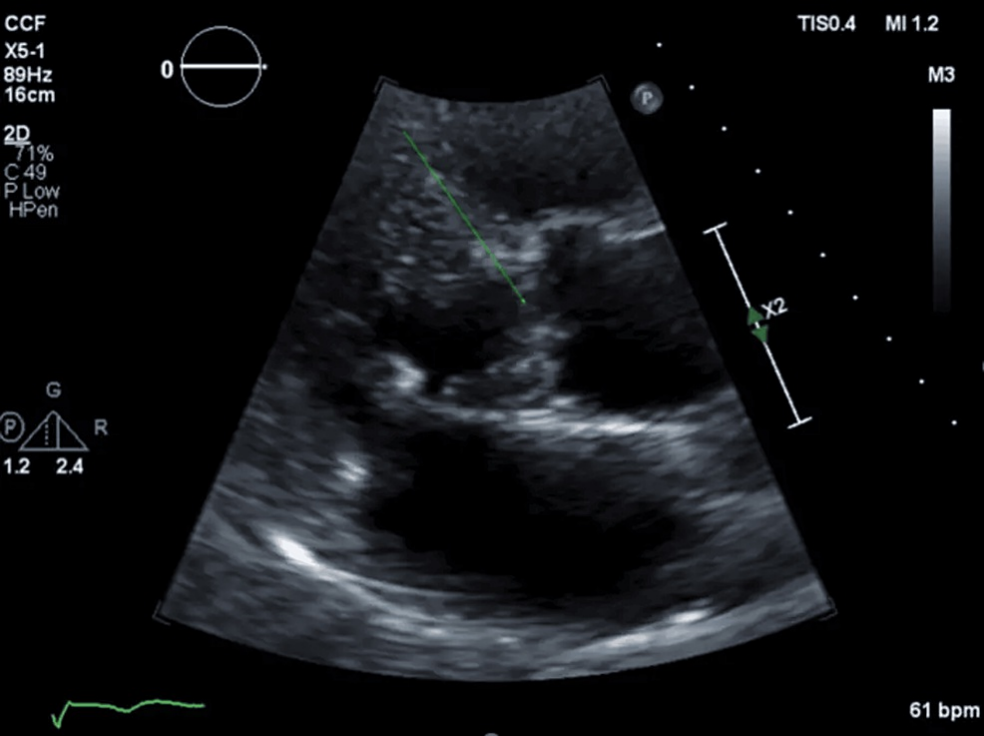
PCI scan Still image from TTE that occurred before PCI. Green arrow shows an intact aortic valve. PCI - percutaneous coronary intervention; TTE - transthoracic echocardiogram

An 81-year-old male patient (177.8 cm height - 94.9 kg weight) with a past medical history of hereditary hemochromatosis (148 ug/dL serum iron, 144.1 ng/dL ferritin, 60.7% transferrin saturation, no pigmentation) requiring biannual phlebotomy, syncope, hyperlipidemia managed with atorvastatin, and hypertension controlled with benazepril (10 mg). He presented to the emergency room for burning chest pain and shortness of breath upon waking and subsequently had a syncopal episode with a fall during a bowel movement. He had undergone a diagnostic workup after his previous syncopal episodes with no notable etiology found. The Emergency Department workup included an unremarkable electrocardiogram (EKG) and mildly elevated high-sensitivity troponins. Additional troponin T levels were elevated, indicative of non-ST-elevation myocardial infarction (NSTEMI). Computed tomography (CT) brain, ventilation/perfusion (V/Q) scan, CT neck, and bilateral deep vein thrombosis (DVT) ultrasounds were all unremarkable. Transthoracic echocardiogram (TTE) revealed an ejection fraction of 58%, grade II left ventricular diastolic dysfunction, trace aortic valve regurgitation, right ventricular dilation, mild tricuspid regurgitation, and right ventricular systolic pressure of 35 mmHg and intact aortic valve (Figure 1). The cardiology team evaluated the patient and elected to perform left heart catheterization the day after admission.

**Figure 2:**
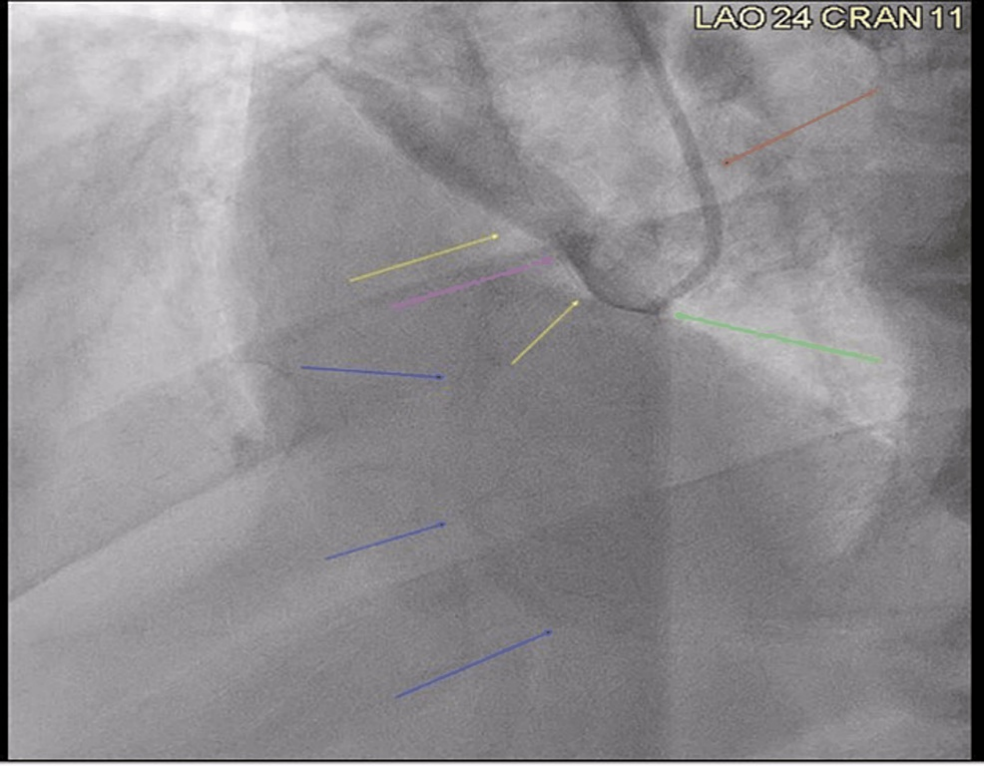
PCI scan Still image from PCI. Red arrow: catheter descending from the aorta with dye flowing through it. Green arrow: tip of the catheter near aortic valve location. Pink arrow: right coronary ostia. Blue arrows: right coronary artery with very faint amount of dye entering the artery. Yellow arrows: large amount of dye blowing back into the aorta. PCI - percutaneous coronary intervention

To initiate the left heart catheterization, access was obtained from the left radial artery using a 5 French sheath. However, attempts to engage the right coronary ostium using the Judkins Right 4.0 (JR4) catheter were unsuccessful. Additional attempts to enter the right coronary ostium using Williams Posterior Right, multipurpose, and Amplatz Right 2 (AR 2) catheters also failed. Next, the decision was made to access the right femoral artery using a 6 French sheath. Despite this new access point, providers were still unable to access the right coronary ostium using both JR4 catheter and AR 1 catheter. Finally, success was finally obtained in entering the right coronary ostium using an AR 2 catheter (Figure 2).

Throughout the remainder of the procedure, triple vessel disease was discovered. Findings included heavy calcifications and stenotic disease in the proximal and middles of the left anterior descending artery, subtotal ostial occlusion of the first diagonal artery, subtotal ostial occlusion of the circumflex artery, and near-total ostial occlusion of the right coronary artery.

No intervention was performed at the time of the left heart catheterization, and the patient was transferred to another facility prior to receiving CABG surgery. He was placed on a heparin infusion, metoprolol 12.5 mg twice a day (BID), acetylsalicylic acid (ASA) 81 mg, isosorbide mononitrate 30 mg, atorvastatin 80 mg, benazepril, and continuous telemetry monitoring. The day after the left heart catheterization, an EKG showed normal sinus rhythm with a heart rate of 59 beats per minute. The patient was managed medically while awaiting CABG. During this time, only oral isosorbide mononitrate was replaced with sublingual nitroglycerin due to the patient's intolerance of isosorbide mononitrate. The patient remained stable during this period with no residual symptoms of coronary artery disease.

**Figure 3:**
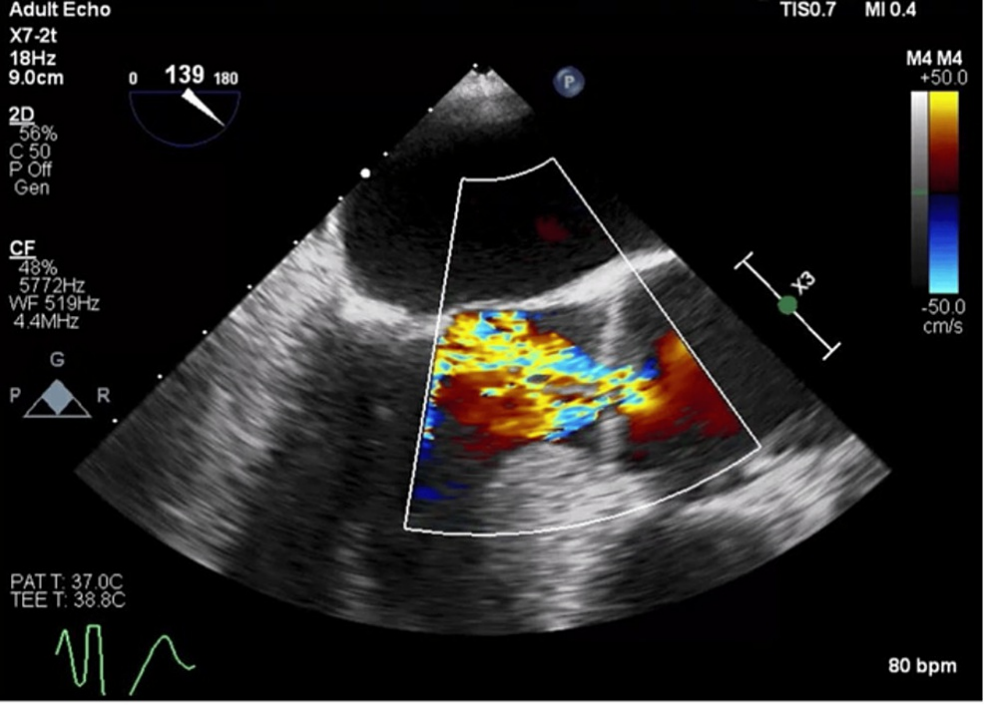
New aortic insufficiency New aortic insufficiency was found on intraoperative TTE prior to CABG and aortic valve repair and replacement. TTE - transesophageal echocardiogram; CABG - coronary artery bypass graft surgery

**Figure 4:**
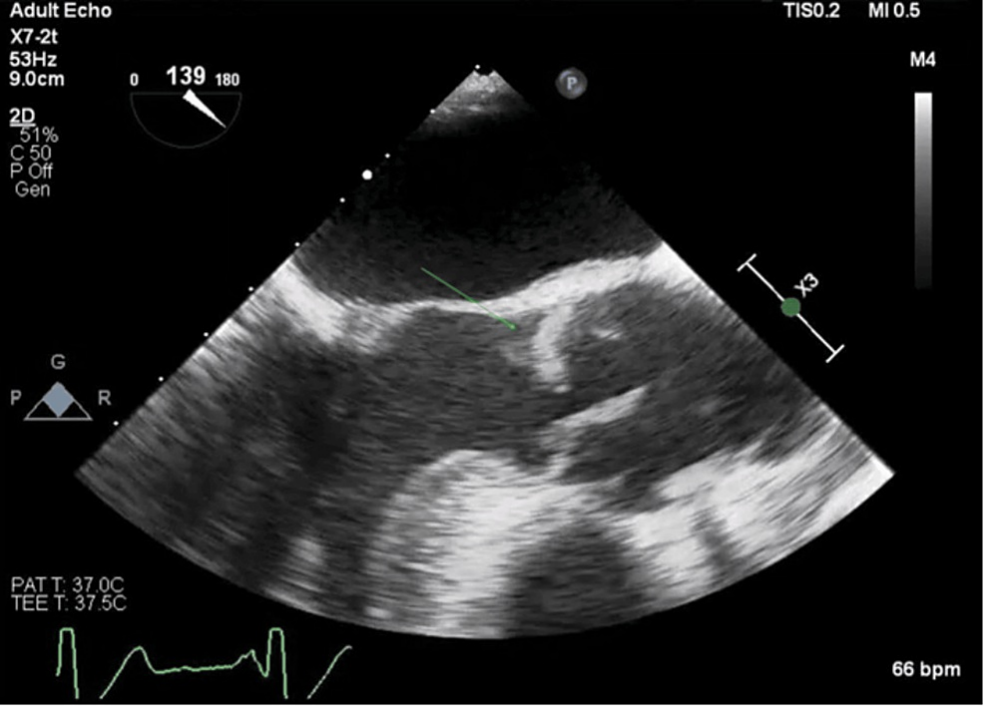
Mobile mass AV LAX view showing mobile mass attached to AV during systole (green arrow) AV - aortic valve; LAX - long axis

**Figure 5:**
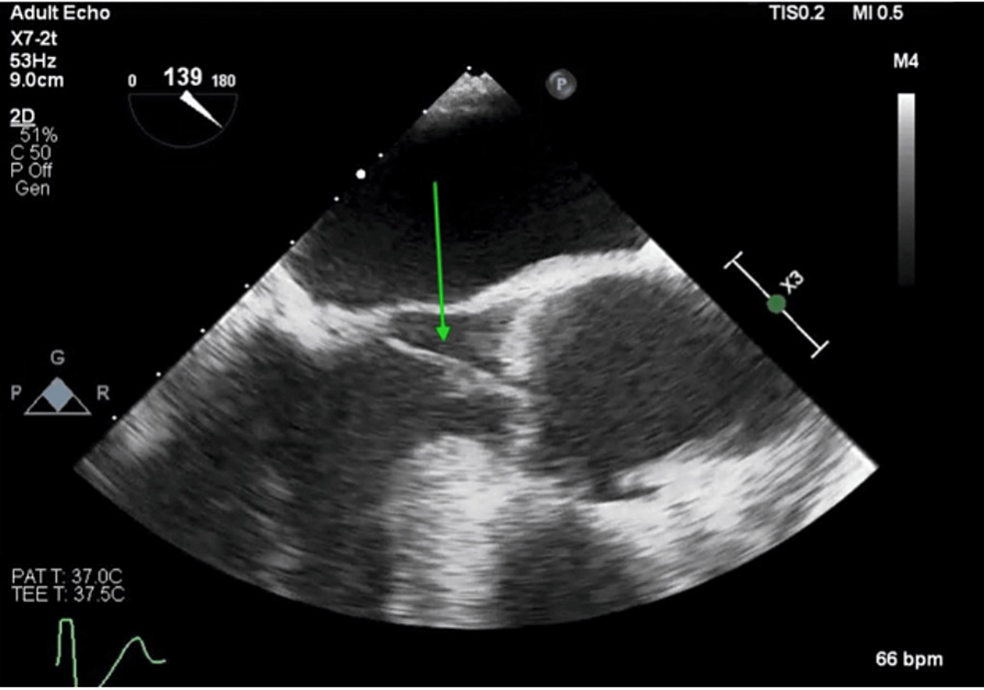
Mobile mass AV LAX view showing mobile mass attached to AV Right coronary cusp prolapsing into LVOT (green arrow) AV - aortic valve; LAX - long axis; LVOT - left ventricular outflow tract

**Figure 6:**
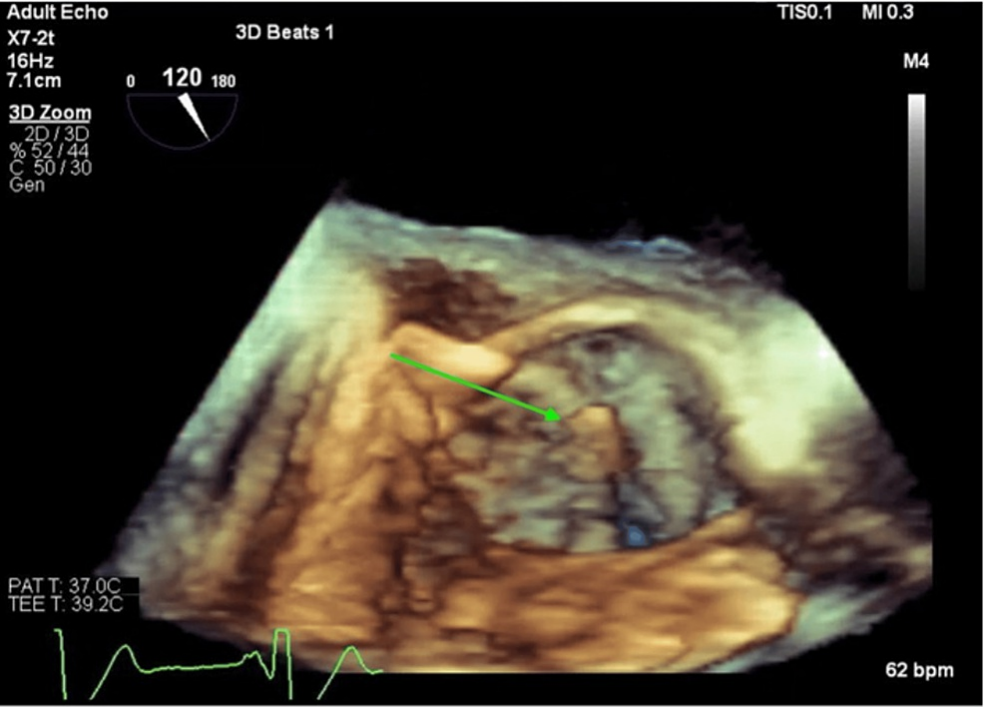
Mobile mass 3D view of mobile mass taken from LVOT (green arrow) LVOT - left ventricular outflow tract

CABG surgery was performed nine days after the initial left heart catheterization. The patient underwent general anesthesia using etomidate, rocuronium, fentanyl, esmolol, and lidocaine. Endotracheal intubation and central line placement were completed without complication. A transesophageal echocardiogram (TTE) revealed new 2+ aortic insufficiency (Figure 3) with a large, mobile echodensity at the right coronary cusp of the aortic valve (Figures 4-6). Moderate concentric left ventricular hypertrophy and borderline aortic dilation were also found. This finding was in stark contrast to the previous transthoracic echocardiogram 10 days prior, which showed only trace aortic regurgitation without a mobile echodensity (Figure 1). 

The surgeon grossly evaluated the aortic valve and identified a complete tear of the right half of the free edge of the aortic valve's right cusp from the leaflet. The surgery team attempted to repair the torn fragment by suturing it back to the leaflet. The grafting procedure continued with the connection of the left internal mammary artery (LIMA) to the 1mid left anterior descending artery (LAD), right saphenous vein graft (rSVG) to the posterior descending artery (PDA), and rSVG to first obtuse marginal artery (OM1). After grafting was completed, the cross-clamp was removed. While attempting to wean from bypass, epinephrine was changed to norepinephrine, then later increased. A transesophageal echocardiogram performed by the anesthesiologist showed residual 2+ aortic insufficiency, and the decision was made to replace the aortic valve.

Following the placement of an Inspiris size #27 prosthetic aortic valve (Edwards Lifesciences, Irvine, California), the transesophageal echocardiogram indicated no aortic regurgitation. The maximum velocity noted was 195.0 cm/s with a peak gradient of 15 mmHg and a mean gradient of 7 mmHg. The patient tolerated being weaned off the pump. As a precautionary measure, a single right ventricle pacing wire was placed, and the sternum was closed. The patient was transferred to the ICU intubated and supported by infusions of norepinephrine and vasopressin.

Postoperatively, the patient recovered in the ICU and was extubated without complications the day after surgery. On postoperative day one, he required temporary administration of epinephrine, 3.5L crystalloid bolus, and 500 mL albumin for blood pressure support. A unit of packed red blood cells (pRBC) was transfused for a Hgb of 9.3 g/dL because his baseline was approximately 14.0 g/dL from hereditary hemochromatosis. On postoperative day two, he required oral metoprolol 12.5 mg BID and amiodarone infusion after developing paroxysmal atrial fibrillation, possibly related to iron overload cardiomyopathy due to hemochromatosis [3]. On postoperative day three, he remained in atrial fibrillation, and digoxin was added. Also, his hemoglobin decreased to 8.5 g/dL, resulting in an additional pRBC transfusion. On postoperative day four, he converted to sinus rhythm, and his amiodarone infusion was transitioned to oral dosing of 400 mg BID. His norepinephrine and vasopressin infusion were also stopped. On postoperative day five, he had several occurrences of paroxysmal atrial fibrillation, which were managed with IV metoprolol 5 mg to convert to sinus rhythm. On postoperative day six, oral metoprolol was increased to 25 mg BID, and apixaban was started. On postoperative day seven, the patient remained in sinus rhythm and was discharged from the ICU to the regular nursing floor. On the regular nursing floor, he remained in sinus rhythm and was discharged home on amiodarone 200 mg BID with plans to follow up with electrophysiology three to four weeks after discharge.

## Discussion

Experiencing acute aortic regurgitation (AR) after coronary angiography or percutaneous coronary intervention (PCI) is an exceedingly rare complication with an incidence of approximately 1 in 10,000 [1]. Only four case reports of acute aortic regurgitation following coronary angiography or percutaneous coronary intervention were identified in the last five years [1,4-6]. The other case reports attribute the cause of acute aortic regurgitation to a perforation or laceration of one of the aortic valve cusps by the catheter or wire during attempts to access the right coronary ostium [1]. In these cases, proceduralists often require different catheters to access the right coronary artery, with a common catheter being the AR 2 [1,4-6]. Most cases, including the present, resolved this complication by the replacement of the aortic valve [1,4-6]. In our case, the surgery team attempted to repair the aortic valve; however, the valve continued to have significant aortic regurgitation, requiring its replacement. In five other cases, surgeons were able to successfully repair the valve [1]. Our case and others also demonstrate the utility of using perioperative echocardiography during CABG or other open heart surgery to assess cardiac anatomy and function, guide the surgical procedure, and evaluate its success. In our case, the patient's aortic regurgitation did not have a relationship with hemochromatosis, did not cause specific symptoms preoperatively, and went undiscovered for nine days. It is possible that the patient's 2+ aortic regurgitation could have been heard on close auscultation during this time, but numerous providers did not report hearing a murmur on physical exams during the nine-day window. This further demonstrates the need for perioperative echocardiography with its increased sensitivity in detecting numerous pathologies, including valvulopathy, that could affect surgical outcomes.

## Conclusions

Acute and severe aortic regurgitation may arise as a result of iatrogenic injury during coronary angiography, particularly when there is damage to one of the aortic valve leaflets. This underscores the critical role of transesophageal echocardiography (TEE) in the diagnostic process. The utilization of TTE is paramount for accurately identifying and managing aortic regurgitation, especially in cases where symptoms are absent and audible murmurs are not detected upon auscultation. This diagnostic approach becomes particularly crucial when suspected complications arise post-coronary angiography or during perioperative open-heart surgery. Surgical intervention is often necessary for resolving aortic regurgitation, and this can involve either valve repair or, more commonly, valve replacement. Such interventions are imperative for restoring proper cardiac function and preventing further complications.
